# Epigenetic hypomethylation and upregulation of NLRC4 and NLRP12 in Kawasaki disease

**DOI:** 10.18632/oncotarget.24851

**Published:** 2018-04-10

**Authors:** Ying-Hsien Huang, Mao-Hung Lo, Xin-Yuan Cai, Ho-Chang Kuo

**Affiliations:** ^1^ Department of Pediatrics, Kaohsiung Chang Gung Memorial Hospital and Chang Gung University College of Medicine, Kaohsiung, Taiwan; ^2^ Kawasaki Disease Center, Kaohsiung Chang Gung Memorial Hospital, Kaohsiung, Taiwan; ^3^ Department of Pediatrics, Chiayi Chang Gung Memorial Hospital, Puzih-City, Taiwan

**Keywords:** NLRC4, NLRP12, Kawasaki disease

## Abstract

**INTRODUCTION:**

Kawasaki disease (KD) is a type of childhood febrile systemic vasculitis. Inflammasomes control inflammatory signaling and are related with the development of KD. In this study, we performed a survey of transcripts and global DNA methylation levels of inflammasome sensors of NOD-like receptors (NLRs) and the downstream interleukin 1β (IL-1β).

**MATERIALS AND METHODS:**

In this study, for the chip studies, we recruited a total of 18 KD patients, who we analyzed before receiving intravenous immunoglobulin (IVIG) and at least 3 weeks after IVIG treatment, as well as 36 non-fever controls by Illumina HumanMethylation 450 BeadChip and Affymetrix GeneChip® Human Transcriptome Array 2.0. A separate group of 78 subjects was performed for real-time quantitative PCR validations.

**RESULTS:**

The expressions of mRNA levels of NLRC4, NLRP12, and IL-1β were significantly upregulated in KD patients compared to the controls (p<0.05). Once KD patients underwent IVIG treatment, these genes considerably decreased. In particular, the methylation status of the CpG sites of these genes indicated a significant opposite tendency between the KD patients and the controls. Furthermore, mRNA levels of IL-1β represented a positive correlation with NLRC4 (p=0.002). We also observed that the mRNA levels of NLRP12 were lower in KD patients who developed coronary arterial lesions (p<0.005).

**CONCLUSION:**

This study is among the first to report epigenetic hypomethylation, increased transcripts, and the upregulation of NLRC4, NLRP12 and IL-1β in KD patients. Moreover, a decreased upregulation of NLRP12 was related to coronary arterial lesion formation in KD patients.

## INTRODUCTION

Kawasaki disease (KD), also known as mucocutaneous lymph node syndrome or infantile periarteritis nodosa, inflames the walls of both small- and medium-sized blood vessels (vasculitis), particularly coronary arteries, throughout the body. In general, KD is found in children under the age of 5 years old [1]. KD’s most serious cardiovascular complications are caused by coronary artery lesions (CALs) and include coronary artery fistula formations [2], arterial remodeling, and coronary artery aneurysms (CAAs) [3]. Although this disease can be treated, nearly 20% of children who do not receive treatment suffer a CAA [2], which, in severe cases, may even cause death.

Toll-like receptors (TLRs) function as the sensor arm of the innate immune system and induce proinflammatory cytokine expressions [4]. We previously found that TLRs, particularly TLR1, 2, 4, 6, 8, and 9, were capable of stimulating the immunopathogenesis of KD [5]. Activating the inflammasome is the key function facilitated by the innate immune system [6]. Furthermore, growing evidence has linked inflammasomes to various autoinflammatory diseases [6], and our research team also provided evidence that KD is an autoimmune-like disease [7]. In recent years, researchers have strongly suggested that autoinflammatory diseases are disorders of the innate immune system, are characterized by systemic inflammation often caused by inflammasomes, and are free from infection and autoreactive antibodies or antigen-specific T cells [8]. NOD-like receptors (NLRs) are intracellular sensors of exogenous pathogens and endogenous damage-associated molecular pattern [9]. Once inflammasomes are activated by NLRs, the activation of caspase-1 is activate to control the expression of such inflammatory cytokines as interleukin 1β (IL-1β) and IL-18 [10]. Furthermore, several clinical and experimental animal models have strongly implicated the function of IL-1β in KD [11–14].

Epigenetics indicates the DNA methylation and acetylation pattern of the genome and subsequently results in changes in the chromatin structure [15]. Previously, we found in another study that treatment with IVIG drastically altered methylation patterns in KD patients [5, 16, 17]. KD patients showed considerably increased mRNA expressions in TLRs and hypomethylation at the gene promoters of TLRs [5], and IVIG treatment can restore the methylation level of TLRs and decrease their mRNA expression [5]. However, no studies have yet surveyed the global gene expressions and methylation profiles in the NLRs of KD patients. Therefore, we aimed to comprehensively examine the mRNA expressions of these genes and analyze methylation level changes in two different stages of KD patients, as well as in control subjects.

## RESULTS

### Differential expression of NLRC4 and NLRP12 mRNA levels in KD patients and controls and changes following IVIG treatment

To investigate the transcript expressions of NOD-like receptors (NOD1, 2, NLRC 3-5, and NLRP 1-14) [18], we used Affymetrix GeneChip® Human Transcriptome Array 2.0 to identify their expression levels. As shown in Table 1, KD patients demonstrated differential expression of NOD-like receptors when compared to both the healthy and febrile control subjects. The mRNA levels of NLRC4 and NLRP12 were significantly higher in KD patients than in the healthy control and febrile control groups. These NLRC4 and NLRP12 values significantly decreased, while NOD-1 significantly increased in KD patients after receiving IVIG treatment (Table 1). However, we found no remarkable differences in the remaining NLRCs and NLRPs among the groups or in the KD patients after undergoing IVIG treatment.

**Table 1:** Transcripts expressions of nucleotide-binding oligomerization domain, leucine rich repeat with caspase recruitment domain (NLRCs) and with pyrin domain (NLRPs), interleukin 1 beta and interleukin-18 between Kawasaki disease patients and control subjects

Symbol	RefSeq	Column ID	Fold-Change (KD1 vs. HC)	p value (KD1 vs. HC)	Fold-Change (KD1 vs. FC)	p value (KD1 vs. FC)	Fold-Change (KD3 vs. KD1)	p value (KD3 vs. KD1)
NOD1	NM_006092	TC07001249.hg.1	1.008	0.897	-1.050	0.459	-1.080	0.254
NOD2	NM_022162	TC16000442.hg.1	1.248	0.066	1.154	0.206	-1.402	0.012^*^
NLRC3	NM_178844	TC16000820.hg.1	-1.187	0.024^*^	-1.136	0.072	1.174	0.031^*^
**NLRC4**	**NM_001199138**	**TC02001723.hg.1**	**4.454**	0.000^*^	**2.159**	0.015^*^	**-4.761**	0.000^*^
NLRC5	NM_032206	TC16000482.hg.1	1.022	0.804	1.015	0.859	-1.032	0.718
NLRP1	NM_001033053	TC17001059.hg.1	-1.361	0.032^*^	-1.122	0.364	1.338	0.040^*^
NLRP2	NM_001174081	TC19000887.hg.1	-1.052	0.480	-1.226	0.018^*^	-1.002	0.973
NLRP3	NM_001079821	TC01002008.hg.1	-1.040	0.750	1.131	0.325	-1.046	0.715
NLRP4	NM_134444	TC19000913.hg.1	-1.052	0.515	-1.074	0.370	1.103	0.226
NLRP5	NM_153447	TC19000915.hg.1	-1.016	0.803	-1.036	0.580	1.067	0.323
NLRP6	NM_138329	TC11000007.hg.1	1.059	0.448	1.080	0.319	1.026	0.731
NLRP7	NM_001127255	TC19001853.hg.1	1.039	0.604	-1.096	0.231	1.032	0.671
NLRP8	NM_176811	TC19000914.hg.1	1.022	0.668	-1.019	0.715	1.010	0.844
NLRP9	NM_176820	TC19001880.hg.1	1.010	0.865	1.011	0.858	1.069	0.274
NLRP10	NM_176821	TC11001381.hg.1	-1.026	0.764	1.015	0.864	1.070	0.442
NLRP11	NM_145007	TC19001881.hg.1	-1.001	0.986	-1.058	0.371	1.095	0.168
**NLRP12**	**NM_033297**	**TC19001826.hg.1**	**1.466**	0.000^*^	**1.158**	0.022^*^	**-1.496**	0.000^*^
NLRP13	NM_176810	TC19001882.hg.1	-1.019	0.867	-1.004	0.974	1.192	0.138
NLRP14	NM_176822	TC11000143.hg.1	-1.033	0.624	-1.017	0.800	1.097	0.190
**IL-1β**	**NM_000576**	**TC02002219.hg.1**	**1.677**	**0.066**	**2.195**	0.012^*^	**-2.475**	0.006^*^
**IL-18**	**NM_001562**	**TC11002293.hg.1**	**1.411**	0.012^*^	**1.115**	**0.338**	**-1.417**	0.011^*^

KD1: Kawasaki disease before IVIG treatment; KD3: Kawasaki disease > 3 weeks after IVIG treatment; FC: febrile control; HC: healthy control.

### Significantly altered CpG sites on NOD-like receptors between KD patients and controls

**Figure 1:**
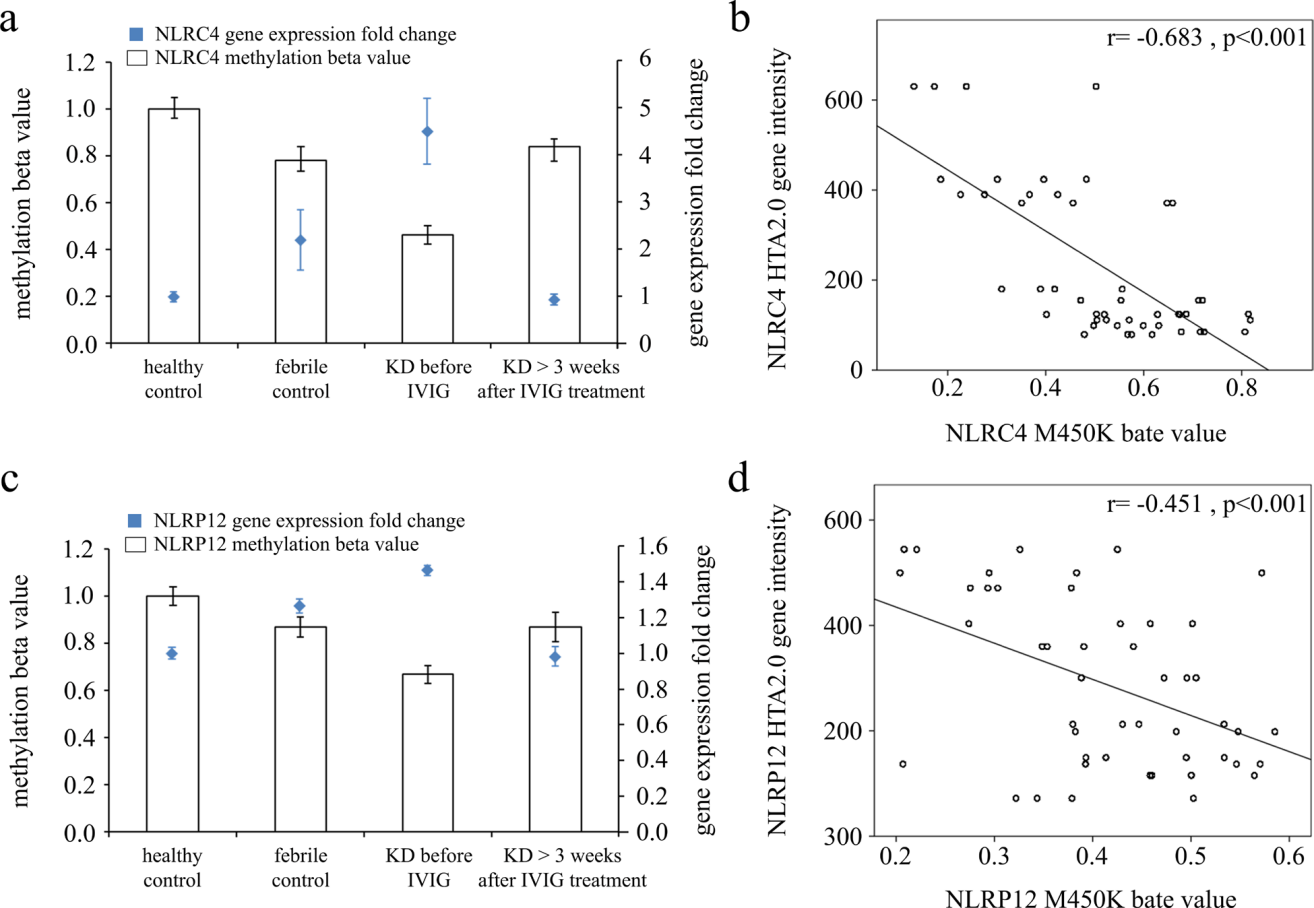
Integration of CpG marker methylation patterns and gene expression profiles of NLRC4 and NLRP12 The methylation patterns of the representative CpG markers (**(a)** cg07055315 for NLRC4 and **(c)** cg22337438 for NLRP12) and gene expression profiles showed negative tendencies and were observed to change in both the healthy and febrile control subjects, as well as KD patients before and after undergoing intravenous immunoglobulin treatment. The histogram and curve are presented as mean ± standard error. **(b, d)** We adopted scatter plots to represent the relationship between mRNA levels and DNA methylation, which demonstrate that mRNA levels were negatively correlated with DNA methylation (Pearson's correlation coefficient approximately -0.683 and -0.451, all p< 0.001).

We adopted Illumina HumanMethylation450 BeadChip (Illumina) to evaluate CpG site methylation patterns on NLRCs and NLRPs between KD patients and both febrile and healthy control subjects (Supplementary Table 1). We found that the NLRC and NLRP methylation levels varied considerably in patients in the acute stage of KD compared to the healthy and febrile controls (Table 2). Furthermore, methylation levels were generally significantly lower in acute-stage KD patients compared to the healthy and febrile controls (Supplementary Table 1). Decreased methylation causes greater gene expression [16], so we focused on the relationship between DNA methylation patterns and gene expressions. Figure 1 shows that both NLRC4 (a) and NLRP12 (b) demonstrate a hypo-methylated status in KD patients that have not yet been treated with IVIG compared to the control subjects and the KD patients already treated with IVIG. Consequently, the mRNA expression level and DNA methylation of NLRC4 and NLRP12 have a negative correlation, which suggests that DNA methylation can repress gene expression.

**Table 2:** Basal characteristics of controls and patients with Kawasaki disease (KD)

Characteristic	Healthy controls (n=18)	Febrile controls (n=53)	Patients with KD (n=79)
Male gender	50%	57%	68%
Mean (SD), age (y)	2.8±1.5	2.6±1.2	1.8±1.6
Age range (y)	1-6	0-5	0-9

### Upregulated transcripts with epigenetic hypomethylation of IL-1β among KD patients and controls and changes following IVIG treatment

**Figure 2:**
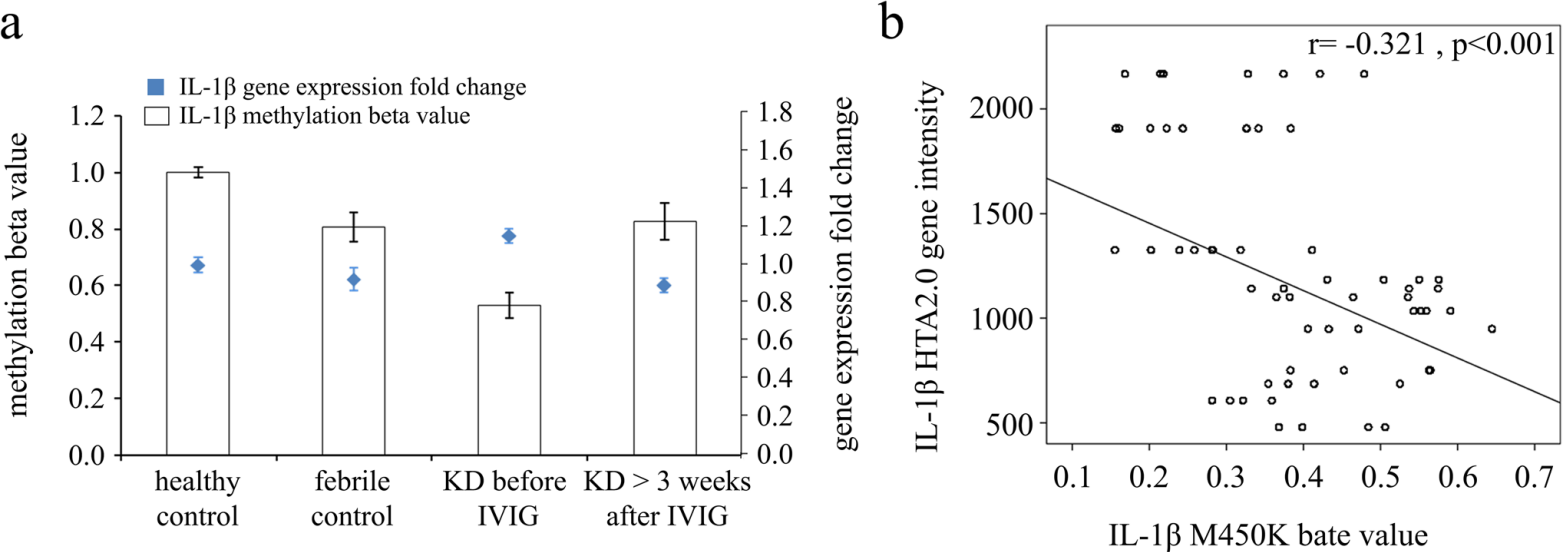
Integration of CpG marker methylation patterns and gene expression profiles of IL-1β **(a)** The methylation patterns of the representative CpG marker and gene expression profile of IL-1β showed negative tendencies and were observed to change in both the healthy and febrile control subjects, as well as KD patients before and after undergoing intravenous immunoglobulin treatment. The histogram and curve are presented as mean ± standard error. **(b)** We adopted scatter plots to represent the relationship between mRNA levels and DNA methylation, which demonstrate that mRNA levels were negatively correlated with DNA methylation (Pearson's correlation coefficient approximately -0.321 and p< 0.001).

We explored whether inflammasome activation was capable of eliciting the expression of downstream proinflammatory cytokines IL-1β. The mRNA levels of IL-1β were significantly elevated in KD patients compared to the healthy control and febrile control groups (Table 1). Furthermore, the mRNA expression level and DNA methylation of IL-1β have a negative correlation, which suggests that DNA methylation is able to repress gene expression (Figure 2).

### NLRC4 and NLRP12 expressions in the peripheral white blood cells (WBCs) of KD patients and controls

**Figure 3:**
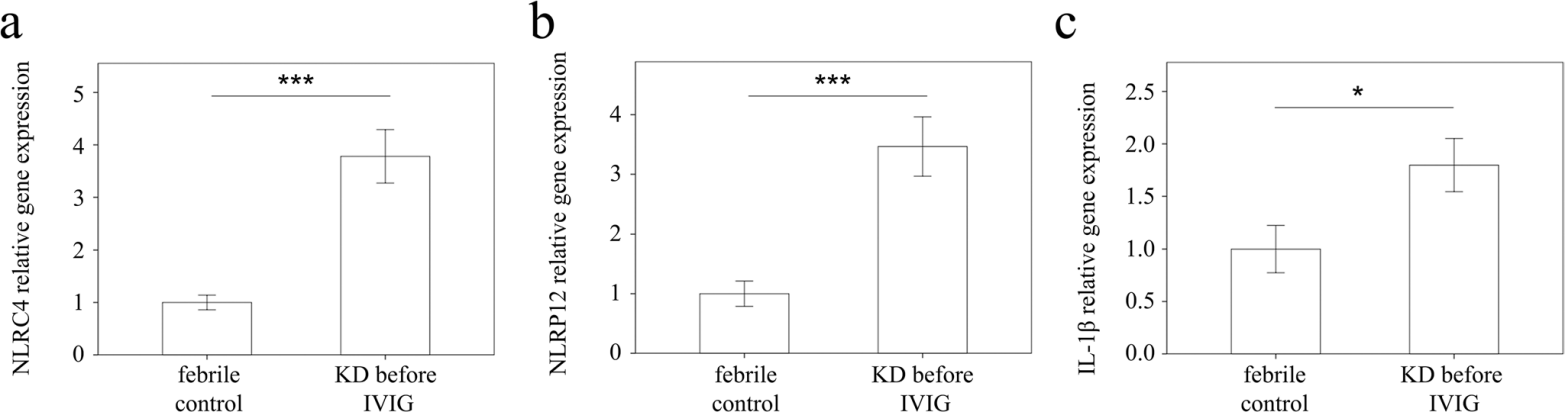
Analyses of **(a)** NLRC4, **(b)** NLRP12, and **(c)** IL-1β mRNA in the peripheral blood mononuclear cells of KD patients (n = 43) and controls (n = 35) using a real-time quantitative polymerase chain reaction. Data are expressed as mean ±standard error. ^*^indicates p < 0.05 and ^***^indicates p < 0.001 between the groups.

**Figure 4:**
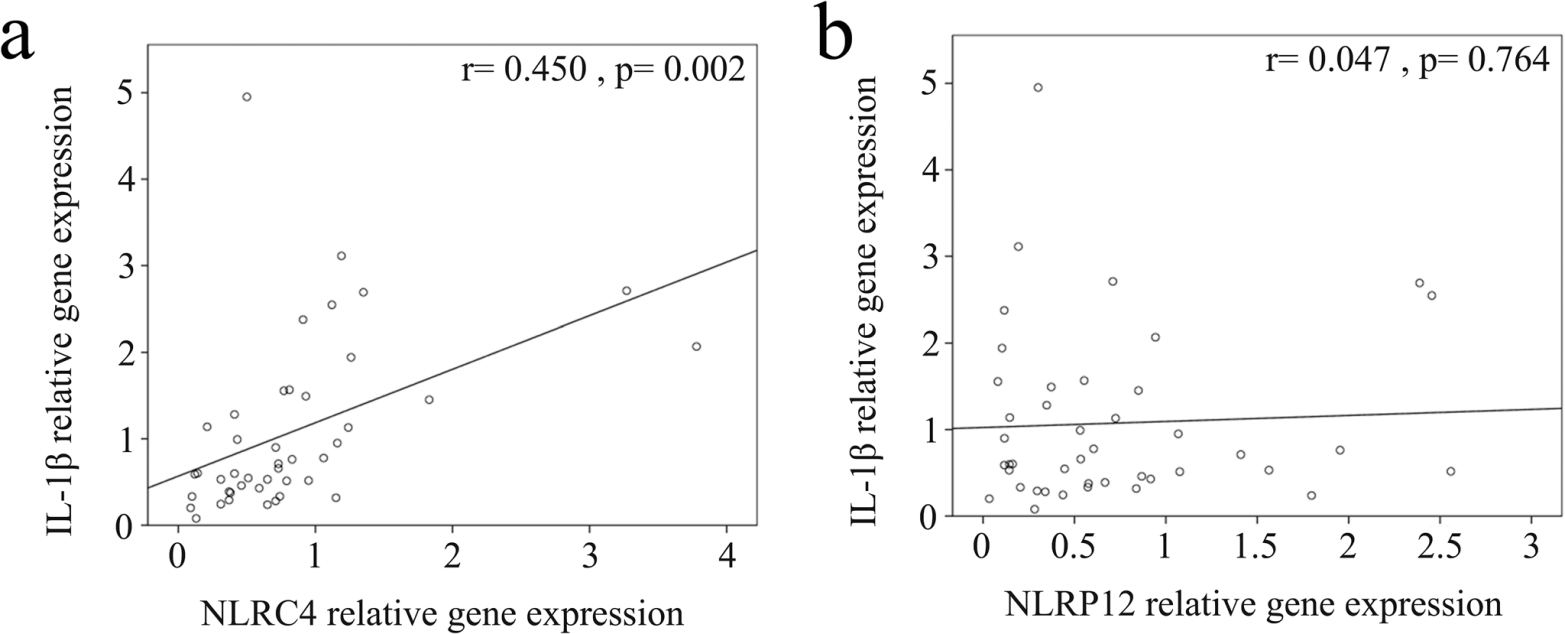
Correlation plots between **(a)** NLRC4 and **(b)** NLRP12 and IL-1β mRNA levels. The mRNA levels of IL-1β have a positive correlation with NLRC4 expression.

**Figure 5:**
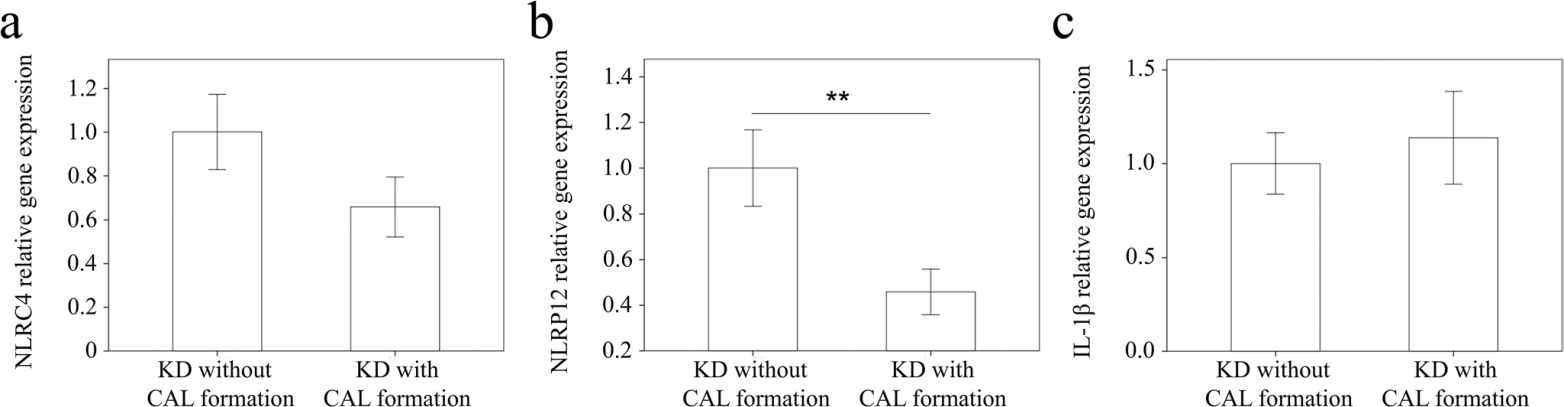
Comparison of **(a)** NLRC4 and **(b)** NLRP12 and **(c)** IL-1β mRNA levels in KD patients without (n =20) and with (n =23) coronary artery lesion (CAL) before being treated with intravenous immunoglobulin. Data are presented as mean ±standard error. ^**^indicates p < 0.01 between the groups.

We used real-time PCR to investigate the mRNA levels of NLRC4, NLRP12, and IL-1β in a separate cohort of 43 KD patients and 35 febrile controls. In doing so, we found elevated NLRC4 (p <0.001), NLRP12 (p <0.001), and IL-1β mRNA levels (p = 0.021) in the WBCs of KD patients compared to those of the controls, as shown in Figure 3. These findings agree with the results obtained with Affymetrix GeneChip® Human Transcriptome Array 2.0. Furthermore, the mRNA level of IL-1β positively correlates with NLRC4 but not NLRP12, which suggests that NLRC4 was associated with IL-1β genes expression in KD patients (Figure 4). Moreover, the NLRP12 mRNA level was lower in KD patients who developed CAL than those who did not (p = 0.0067) (Figure 5).

## DISCUSSION

As far as we know, our study is the first to perform a comprehensive survey of global DNA methylation levels and transcripts of inflammasome sensors of the NOD-like receptors between KD patients and control subjects. Our observations of particular interest include the epigenetic hypomethylation and upregulation of NLRC4 and NLRP12 in KD, as well as the increase of the downstream inflammatory cytokine IL-1β in KD patients. Furthermore, IL-1β expression levels positively correlate with NLRC4, while NLRP12 was lower in KD patients who developed CAL than those who did not.

Growing evidence has suggested that cytokine profiles are associated with the pathogenesis of KD, but the actual CALs involved are still unclear. In our previous studies, we found that KD patients demonstrated considerably increased mRNA expression in TLRs and hypomethylation at TLR gene promoters [5]. Inflammasomes are multimeric protein complexes that gather in cellular cytosol after sensing innate immune system receptors and sensors in response to external infectious microbes or molecules from host proteins [6]. Activating inflammasome signaling is a key part in the pathogenesis of various cardiovascular disorders, including coronary ischemia, cardiomyopathy, and KD [19]. In this study, we have discovered significant epigenetic hypomethylation, increased transcripts, and the upregulation of the NLRC4 and NLRP12 inflammasome sensors. Our study is in line with that of Ikeda et al., who also found up-regulated NLRC4 in acute-phase KD in microarray study [20]. Not only is NLRC4 involved in sensing more than one bacterial molecule, but it also participates in a number of immune complexes [21]. Kitamura et al. identified a missense mutation in NLRC4 in patients with familial cold autoinflammatory syndrome, which promoted the formation of NLRC4-containing inflammasomes that cleave procaspase-1 and increase IL-1β production in order to promote the genesis of inflammatory diseases [22]. Another study identified that the somatic mutation of NLRC4 causes autoinflammatory symptoms that are compatible with neonatal-onset multisystem inflammatory disease [23].

NLRP12 is a cytoplasmic sensor that can be categorized as a negative regulator of inflammation [24] and is related to autoimmune diseases [25, 26]. Meanwhile, NLRP12 mutations cause hereditary periodic fever syndromes [27] and systemic autoinflammatory diseases [28]. Of particular note, Jin et al. observed that decreased NLRP12 in heart tissue from PM2.5 -induced mice was associated with cardiac inflammatory injury [29]. Furthermore, NLRP12 has anti-inflammatory function and down-regulation of NLRP12 is required for dextran sodium sulfate -induced release of proinflammatory cytokinesIL-1β [30]. Likewise, we also found that NLRP12 was lower in KD patients who developed CAL than those who did not, as well as that IL-1β mRNA levels do not have a positive correlation with NLRP12. Both of these findings support the theory that NLRP12 functions as a negative regulator of inflammation in KD patients. Based on the findings, we may apply NLRC4 and NLRP12 gene expressions and DNA methylation as KD biomarkers and develop a high-performance KD diagnosis model.

Activated macrophage produces IL-1β, a master cytokine of systemic inflammation, which has a vital function in auto-inflammatory diseases [31, 32] and has been more recently connected to KD vasculitis [11–13]. Maury et al. found that the serum level of IL-1β is significantly increased in KD patients [33]. IL-1β polymorphism has been associated with KD susceptibility [34] and IVIG resistance in Taiwanese children with KD [35]. Furthermore, IL-1β has been shown to induce myocarditis and coronary aneurysm formation in the Lactobacillus casei cell-wall extract mouse model of KD [12, 13]. In consistence with our findings in KD [36, 37], IL-1β pathway stimulation leads to excess production of hepcidin, which could be causative to anemia of inflammation [38]. In fact, three clinical trials of IL-1 blockade enrolling KD patients are currently being conducted in the U.S. and Europe, and these studies may be able to change the outcome of KD [39].

## MATERIALS AND METHODS

### Patients

In this study, we analyzed the DNA methylation levels of NLRs in 18 KD patients, before treatment and after at least 3 weeks from receiving intravenous immunoglobulin (IVIG), and in 36 healthy (non-fever) controls by Illumina HumanMethylation 450 BeadChip and Affymetrix GeneChip® Human Transcriptome Array 2.0. Subsequently, we validated the mRNA levels of NLRs in 43 KD patients and 35 febrile subjects by real-time quantitative PCR (Table 2). KD patients met the American Heart Association’s diagnosis criteria for KD, which is characterized by extended fever for more than five days, conjunctivitis, diffuse mucosal inflammation, polymorphous skin rashes, indurative edema of the hands and feet associated with peeling of the finger tips in the subacute stage, and non-suppurative lymphadenopathy [40, 41], and treated with once dose of high-dose IVIG treatment (2 g/kg) over 12 h at our hospital. The patients in the fever control group had diagnoses of acute tonsillitis, croup, acute bronchitis, bronchopneumonia, acute sinusitis, or urinary tract infection. We took peripheral blood samples from KD patients prior to undergoing IVIG treatment (pre-IVIG) and then at least three weeks after completing the IVIG treatment, as previously described in another of our studies [42]. A CAL was defined as a coronary artery with an internal diameter of at least 3 mm (4 mm if the patient was older than 5 years) or a segment with an internal diameter at least 1.5 times larger than that of an adjacent segment, as observed through echocardiography [43, 44]. This study was approved by the Chang Gung Memorial Hospital’s Institutional Review Board (IRB No.:101-4618A3), and we obtained written informed consent from the parents or guardians of all subjects. All of the methods that we used complied with the relevant guidelines established.

### Experiment design

We first collected whole blood samples from the subjects and submitted them to WBC enrichment, as previously described in another study [16]. The enriched WBC samples were then subjected to either RNA or DNA extraction. In accordance with the manufacturer’s instructions, we used an isolation kit (mirVana™ miRNA Isolation Kit, Catalog number: AM1560, Life Technologies, Carlsbad, CA) to isolate the total RNA and then calculated both the quality (RIN value) and quantity of the RNA samples using Bioanalyzer (ABI) and Qubit (Thermo). All RNA samples passed the criterion of RIN≧7. We isolated the DNA samples and treated them with bisulfite as previously described in another study [45].

### Gene expression profiling with microarray

For strong, unbiased results, we created pooled RNA libraries by evenly pooling six RNA samples, resulting in three pooled healthy control, three fever control, three pre-IVIG, and three post-IVIG libraries. We performed microarray assay on the pooled RNA samples in order to establish the gene expression profiles and then further performed profiling with GeneChip® Human Transcriptome Array 2.0 (HTA 2.0, Affymetrix, Santa Clara). We used the WT PLUS Reagent kit to prepare the RNA samples and carry out hybridization on the HTA 2.0 microarray chips. Following the Affymetrix instruction manual, we subjected the HTA 2.0 chips’ raw data to quality control examination, as previously described in another study [5].

### DNA methylation profiling with Illumina M450K BeadChip

We adopted Illumina HumanMethylation450 (M450K) BeadChip to perform genome-wide screening of DNA methylation patterns. The M450K BeadChip program was created to detect methylation patterns of approximately 450,000 CpG markers and thus spans the entire human genome. More information about M450 BeadChip can be found at the following website: http://support.illumina.com/array/array_kits/infinium_humanmethylation450_beadchip_kit.html. For each M450K BeadChip assay, we applied 200 ng of bisulfite-converted genomic DNA pursuant to the manufacturer’s instructions [16]. Then, we calculated the methylation percentage of cytosine for each CpG marker in each sample, which we referred to as the β value.

### RNA isolation and real-time quantitative RT-PCR

To quantify the mRNA levels of NLRC4, NLRP12, and IL-1β, we used the LightCycler® 480 Real-Time PCR System (Roche Molecular Systems, Inc. IN, USA) to carry out real-time quantitative PCR. We separated the total mRNA from the WBC using an isolation kit (mirVana™ miRNA Isolation Kit, Catalog number: AM1560, Life Technologies, Carlsbad, CA) in accordance with the manufacturer’s instructions. We performed PCR using a SYBR Green PCR Master Mix containing 10 μM of specific forward and reverse primers. The relative quantification of gene expression was carried out based on the comparative threshold cycle (C_T_) method, which enabled us to determine the target amount as 2^−(ΔCT target − Δ CT calibrator)^ or 2^−ΔΔCT^ [46]. Primers were designed to amplify the target genes, as shown in Table 3. We performed all experiments twice to verify and validate the amplification efficiencies.

**Table 3:** Primers list

Gene symbol	Accession number	Hybridization	Primers (5’ to 3’)
RNA18S	NR_003286.2	forward	GTAACCCGTTGAACCCCATT
		reverse	CCATCCAATCGGTAGTAGCG
NLRC4	NM_001199138.1	forward	GCCTCAGGCTGCAAATAAAG
		reverse	GGCTTCCACCATGAGAGAATAA
NLRP12	NM_033297.2	forward	GGCTCATGTATGTAATCCTAGCA
		reverse	CGGGTTCAAGCGATTCT
IL-1β	NM_000576	forward	CAAAGGCGGCCAGGATATAA
		reverse	CTAGGGATTGAGTCCACATTCAG

### Statistical analysis

We have presented all data as mean ± standard error. Once chips passed the quality control criteria, we evaluated them with Partek (Partek, St. Louis), which is commercial software specifically designed to analyze microarray data. Using Partek, we conducted ANOVA analysis and reported the p-values of comparisons of interest, as previously described [5]. We adopted Student’s t-test or one-way ANOVA as necessary to evaluate the quantitative data and the paired sample *t*-test to evaluate any data changes before and after IVIG treatment. All statistical analyses were carried out with SPSS version 12.0 for Windows XP (SPSS, Inc., Chicago, USA), and we considered a two-sided p-value less than 0.05 statistically significant.

## CONCLUSIONS

Insights into the mechanisms that govern inflammasome activation in KD will help medical professionals to better understand the pathogenesis of KD. Our study is the first to observe DNA hypomethylation and increased NLRC4 and NLRP12 transcripts in KD compared to both kinds of control subjects. Furthermore, NLRC4 was correlated with the upregulation of IL-1β, while a decreased upregulation of NLRP12 was related to CAL formation in KD patients.

## SUPPLEMENTARY MATERIALS TABLE




