# Supplementary material for: Epigenetic hypomethylation and upregulation of NLRC4 and NLRP12 in Kawasaki disease
Source: Oncotarget. 2018 Apr 10;9(27):18939–48. doi: 10.18632/oncotarget.24851 (PMC5922368; doi:10.18632/oncotarget.24851)
Supplement: Supplementary file 1 [file oncotarget-09-18939-s001.pdf]

Epigenetic hypomethylation and upregulation of NLRC4 and NLRP12 in Kawasaki disease

SUPPLEMENTARY MATERIALS

Supplementary Table 1: Methylation patterns of CpG sites on nucleotide-binding oligomerization domain, leucine rich repeat with caspase recruitment domain (NLRCs) and with pyrin domain (NLRPs), interleukin 1 beta and interleukin-18 between Kawasaki disease patients and control subjects

See Supplementary File 1
