# Supplementary material for: Epigenetic hypomethylation and upregulation of NLRC4 and NLRP12 in Kawasaki disease
Source: Oncotarget. 2018 Apr 10;9(27):18939–48. doi: 10.18632/oncotarget.24851 (PMC5922368; doi:10.18632/oncotarget.24851)
Supplement: Supplementary file 2 [file oncotarget-09-18939-s002.doc]

Supplementary table**:** Methylation patterns of CpG sites on nucleotide-binding oligomerization domain, leucine rich repeat with [caspase recruitment domain](https://en.wikipedia.org/wiki/CARD_domain) (NLRCs) and with pyrin domain (NLRPs) , interleukin 1 beta and interleukin-18 between Kawasaki disease patients and control subjects

| **Target ID** | **Symbol** | **Fold-Change**  **(KD1 vs. HC)** | ***p*-value**  **(KD1 vs. HC)** | **Fold-Change**  **(KD1 vs. FC)** | ***p*-value**  **(KD1 vs. FC)** | **Fold-Change**  **(KD3 vs. KD1)** | ***p*-value**  **(KD3 vs. KD1)** | | |
| --- | --- | --- | --- | --- | --- | --- | --- | --- | --- |
| cg02968854 | NOD1 | 1.001 | 0.14 | -1.002 | 0.015* | 1.004 | | 0.000* |  |
| cg04071779 |  | -1.095 | 0.000* | -1.045 | 0.000* | 1.04 | | 0.001* |  |
| cg11081941 |  | 1.003 | 0.158 | 1.007 | 0.000* | -1.013 | | 0.000* |  |
| cg13047462 |  | -1.004 | 0.005* | -1.005 | 0.000* | 1.005 | | 0.001* |  |
| cg14037546 |  | -1.016 | 0.001* | -1.006 | 0.214 | -1.001 | | 0.895 |  |
| cg15815016 |  | 1.001 | 0.554 | -1.009 | 0.000* | 1.012 | | 0.000* |  |
| cg16391151 |  | -1.001 | 0.396 | -1.002 | 0.215 | 1.007 | | 0.000* |  |
| cg16680624 |  | -1.055 | 0.000* | -1.027 | 0.002* | -1.005 | | 0.527 |  |
| cg18682277 |  | -1.02 | 0.000* | -1.038 | 0.000* | 1.029 | | 0.000* |  |
| cg18789624 |  | -1.008 | 0.151 | 1.009 | 0.091 | -1.065 | | 0.000* |  |
| cg22476897 |  | -1.005 | 0.021* | -1.005 | 0.010* | 1.012 | | 0.000* |  |
| cg22517552 |  | 1.003 | 0.058 | -1.001 | 0.442 | -1.005 | | 0.001* |  |
| cg23378989 |  | -1 | 0.827 | -1.001 | 0.634 | 1.005 | | 0.032* |  |
| cg27376617 |  | 1 | 0.922 | -1.007 | 0.000* | 1.007 | | 0.000* |  |
| cg27392024 |  | 1.001 | 0.721 | 1.001 | 0.607 | 1.003 | | 0.161 |  |
| cg01902066 | NOD2 | -1.109 | 0.000* | -1.077 | 0.000* | 1.042 | | 0.032* |  |
| cg02119982 |  | 1.001 | 0.889 | 1.005 | 0.266 | -1.068 | | 0.000* |  |
| cg06181567 |  | -1.006 | 0.107 | 1.002 | 0.495 | -1.029 | | 0.000* |  |
| cg07038098 |  | 1.002 | 0.425 | -1.002 | 0.3 | 1.013 | | 0.000* |  |
| cg08554257 |  | -1.197 | 0.000* | -1.092 | 0.000* | 1.127 | | 0.000* |  |
| cg09559780 |  | -1.106 | 0.000* | -1.059 | 0.002* | 1.051 | | 0.008* |  |
| cg12800047 |  | -1.006 | 0.45 | -1.013 | 0.099 | -1.01 | | 0.201 |  |
| cg16771652 |  | -1.106 | 0.000* | -1.078 | 0.000* | 1.064 | | 0.000* |  |
| cg18177814 |  | -1.082 | 0.000* | -1.041 | 0.001* | 1.016 | | 0.164 |  |
| cg19351954 |  | 1.005 | 0.758 | 1.002 | 0.914 | 1.001 | | 0.943 |  |
| cg26954174 |  | -1.192 | 0.000* | -1.103 | 0.000* | 1.121 | | 0.000* |  |
| cg02016178 | NLRC3 | 1.009 | 0.007* | 1.017 | 0.000* | -1.026 | | 0.000* |  |
| cg02127172 |  | -1.032 | 0.000* | -1.012 | 0.124 | 1.035 | | 0.000* |  |
| cg03320754 |  | 1.003 | 0.236 | 1.012 | 0.000* | -1.005 | | 0.072 |  |
| cg05886954 |  | -1.006 | 0.202 | 1.004 | 0.433 | -1.04 | | 0.000* |  |
| cg10100747 |  | -1.004 | 0.105 | -1.003 | 0.257 | 1.009 | | 0.001* |  |
| cg12150022 |  | 1.016 | 0.198 | 1.005 | 0.678 | -1.018 | | 0.168 |  |
| cg21615654 |  | -1.05 | 0.006* | -1.015 | 0.397 | -1.002 | | 0.889 |  |
| cg22171142 |  | -1.021 | 0.000* | 1.002 | 0.675 | -1.021 | | 0.000* |  |
| cg27179111 |  | 1.014 | 0.001* | 1.035 | 0.000* | -1.038 | | 0.000* |  |
| cg05057291 | NLRC4 | -1.006 | 0.051 | -1.004 | 0.219 | -1.028 | | 0.000* |  |
| **cg07055315** |  | **-1.272** | **0.000*** | **-1.148** | **0.000*** | **1.181** | | **0.000*** |  |
| cg11762595 |  | -1.006 | 0.08 | 1.006 | 0.071 | -1.046 | | 0.000* |  |
| cg22805603 |  | -1.221 | 0.000* | -1.118 | 0.000* | 1.151 | | 0.000* |  |
| cg04799664 | NLRC5 | -1.005 | 0.354 | 1.004 | 0.388 | -1.047 | | 0.000* |  |
| cg02770097 | NLRP1 | 1 | 0.971 | -1.006 | 0.5 | -1.002 | | 0.859 |  |
| cg03453638 |  | 1.004 | 0.025* | 1.002 | 0.172 | -1.006 | | 0.001* |  |
| cg05716075 |  | -1.004 | 0.233 | 1.002 | 0.631 | -1.004 | | 0.255 |  |
| cg22642477 |  | -1.004 | 0.083 | -1.006 | 0.003* | -1.004 | | 0.047* |  |
| cg22791700 |  | -1.007 | 0.212 | -1.004 | 0.471 | -1.026 | | 0.000* |  |
| cg26561413 |  | -1.002 | 0.564 | -1.009 | 0.001* | 1.007 | | 0.017* |  |
| cg01477633 | NLRP2 | -1.002 | 0.359 | -1.003 | 0.056 | 1.002 | | 0.151 |  |
| cg03187614 |  | 1.006 | 0.937 | 1.011 | 0.888 | 1.022 | | 0.778 |  |
| cg08237707 |  | -1.004 | 0.312 | -1.001 | 0.83 | -1.003 | | 0.422 |  |
| cg08260406 |  | -1.024 | 0.508 | 1.022 | 0.551 | -1.022 | | 0.541 |  |
| cg15561305 |  | -1.013 | 0.202 | 1.004 | 0.724 | 1.012 | | 0.259 |  |
| cg18059223 |  | -1.005 | 0.362 | 1.011 | 0.031* | -1.065 | | 0.000* |  |
| cg19219661 |  | -1 | 0.923 | 1.002 | 0.306 | -1 | | 0.991 |  |
| cg19752722 |  | 1.002 | 0.42 | -1.001 | 0.834 | -1.002 | | 0.553 |  |
| cg27572999 |  | -1.002 | 0.25 | -1 | 0.843 | -1.002 | | 0.364 |  |
| cg03466998 | NLRP3 | -1.203 | 0.000* | -1.092 | 0.000* | 1.105 | | 0.000* |  |
| cg03505654 |  | -1.009 | 0.104 | -1.016 | 0.007* | 1.06 | | 0.000* |  |
| cg07313373 |  | -1.03 | 0.000* | -1.019 | 0.000* | 1.023 | | 0.000* |  |
| cg14413862 |  | -1.009 | 0.003* | -1.001 | 0.852 | -1.018 | | 0.000* |  |
| cg18183941 |  | -1.064 | 0.000* | -1.035 | 0.000* | 1.016 | | 0.066 |  |
| cg21806273 |  | -1.06 | 0.000* | -1.038 | 0.000* | 1.035 | | 0.001* |  |
| cg21824010 |  | -1.105 | 0.000* | -1.062 | 0.000* | 1.091 | | 0.000* |  |
| cg21919599 |  | -1.008 | 0.008* | -1.015 | 0.000* | 1.027 | | 0.000* |  |
| cg21991396 |  | -1.278 | 0.000* | -1.158 | 0.000* | 1.208 | | 0.000* |  |
| cg24639969 |  | -1.104 | 0.000* | -1.091 | 0.000* | 1.151 | | 0.000* |  |
| cg26112639 |  | -1.112 | 0.000* | -1.086 | 0.000* | 1.068 | | 0.000* |  |
| cg00554118 | NLRP4 | -1.005 | 0.525 | 1.006 | 0.459 | -1.121 | | 0.000* |  |
| cg01861389 |  | 1.002 | 0.612 | 1.003 | 0.352 | -1.005 | | 0.083 |  |
| cg14003035 |  | -1.006 | 0.498 | -1 | 0.979 | -1.012 | | 0.191 |  |
| cg14940165 |  | 1.003 | 0.445 | -1.003 | 0.377 | 1.005 | | 0.146 |  |
| cg16185840 |  | -1.002 | 0.585 | -1.001 | 0.873 | -1.012 | | 0.001* |  |
| cg16345566 |  | -1.053 | 0.317 | 1.066 | 0.215 | 1.004 | | 0.942 |  |
| cg17382459 |  | -1.001 | 0.905 | 1.011 | 0.114 | -1.024 | | 0.001* |  |
| cg17856544 |  | -1.012 | 0.008* | -1.006 | 0.164 | -1.057 | | 0.000* |  |
| cg18006085 |  | -1.004 | 0.114 | -1.003 | 0.237 | 1.002 | | 0.431 |  |
| cg21113318 |  | 1.001 | 0.945 | -1.024 | 0.028* | -1.01 | | 0.336 |  |
| cg25730098 |  | -1.024 | 0.000* | -1.012 | 0.012* | 1.007 | | 0.129 |  |
| cg05023116 | NLRP5 | -1.018 | 0.000* | -1.006 | 0.162 | -1.001 | | 0.861 |  |
| cg14976001 |  | -1.003 | 0.001* | 1.003 | 0.000* | -1.003 | | 0.000* |  |
| cg19697575 |  | -1.01 | 0.902 | 1.112 | 0.207 | -1.03 | | 0.722 |  |
| cg19754387 |  | -1.002 | 0.267 | -1.003 | 0.093 | 1.007 | | 0.001* |  |
| cg23985930 |  | -1.005 | 0.477 | 1.004 | 0.601 | -1.129 | | 0.000* |  |
| cg02471658 | NLRP6 | -1.018 | 0.012* | 1.015 | 0.032* | -1.025 | | 0.001* |  |
| cg02925222 |  | -1.016 | 0.004* | -1.033 | 0.000* | 1 | | 0.964 |  |
| cg03971469 |  | -1.009 | 0.7 | 1.006 | 0.795 | -1.034 | | 0.146 |  |
| cg06328831 |  | -1.013 | 0.074 | -1.027 | 0.001* | -1.022 | | 0.005* |  |
| cg09205751 |  | 1.018 | 0.081 | 1.029 | 0.006* | -1.101 | | 0.000* |  |
| cg10152627 |  | -1.035 | 0.048* | -1.005 | 0.748 | -1.02 | | 0.253 |  |
| cg10330545 |  | -1.017 | 0.015* | -1.031 | 0.000* | 1.011 | | 0.113 |  |
| cg15433198 |  | -1.006 | 0.010* | -1.005 | 0.026* | 1.002 | | 0.422 |  |
| cg15451020 |  | 1.014 | 0.009* | 1.016 | 0.005* | -1.037 | | 0.000* |  |
| cg16359142 |  | 1.001 | 0.799 | 1.008 | 0.175 | -1.039 | | 0.000* |  |
| cg18016138 |  | 1.02 | 0.051 | 1.019 | 0.055 | -1.04 | | 0.000* |  |
| cg22331032 |  | 1.025 | 0.005* | 1.015 | 0.077 | -1.089 | | 0.000* |  |
| cg23437420 |  | 1.007 | 0.21 | 1.006 | 0.291 | -1.026 | | 0.000* |  |
| cg23743606 |  | -1.016 | 0.002* | 1.009 | 0.074 | -1.019 | | 0.000* |  |
| cg27120999 |  | -1.014 | 0.083 | -1.023 | 0.006* | -1.001 | | 0.864 |  |
| cg01628819 | NLRP7 | -1.002 | 0.328 | 1.006 | 0.027* | -1.015 | | 0.000* |  |
| cg01879460 |  | -1.006 | 0.263 | -1.012 | 0.023* | -1.016 | | 0.003* |  |
| cg08211199 |  | -1.021 | 0.000* | -1.01 | 0.048* | 1.002 | | 0.622 |  |
| cg13897449 |  | 1.006 | 0.488 | 1.022 | 0.017* | -1.023 | | 0.011* |  |
| cg14876043 |  | -1.007 | 0.247 | 1.005 | 0.392 | -1.055 | | 0.000* |  |
| cg18449187 |  | -1.002 | 0.651 | 1.011 | 0.005* | -1.023 | | 0.000* |  |
| cg27131908 |  | -1.016 | 0.000* | 1.003 | 0.462 | -1.054 | | 0.000* |  |
| cg00751958 | NLRP8 | -1.004 | 0.464 | 1.002 | 0.709 | -1.033 | | 0.000* |  |
| cg01292613 |  | -1.002 | 0.652 | 1.01 | 0.026* | -1.027 | | 0.000* |  |
| cg02241986 |  | -1.009 | 0.286 | 1.009 | 0.274 | 1.014 | | 0.104 |  |
| cg08026930 |  | -1.008 | 0.301 | -1.004 | 0.636 | 1.036 | | 0.000* |  |
| cg26351481 |  | -1.006 | 0.214 | 1.006 | 0.203 | -1.093 | | 0.000* |  |
| cg03149814 | NLRP9 | -1.007 | 0.049* | 1.006 | 0.101 | -1 | | 0.927 |  |
| cg07821737 |  | -1.007 | 0.309 | 1.011 | 0.1 | -1.079 | | 0.000* |  |
| cg08475088 |  | -1.005 | 0.188 | -1.004 | 0.311 | -1.013 | | 0.001* |  |
| cg12766231 |  | -1.009 | 0.019* | -1 | 0.925 | -1.008 | | 0.041* |  |
| cg24819427 |  | -1.005 | 0.453 | 1.022 | 0.001* | -1.139 | | 0.000* |  |
| cg03353436 | NLRP10 | -1.017 | 0.000* | -1.008 | 0.008* | -1.011 | | 0.001* |  |
| cg06679185 |  | -1.02 | 0.000* | -1.006 | 0.219 | -1.013 | | 0.014* |  |
| cg07572949 |  | -1.009 | 0.307 | 1.01 | 0.252 | -1.025 | | 0.004* |  |
| cg16560627 |  | -1.018 | 0.003* | -1.002 | 0.672 | -1.012 | | 0.043* |  |
| cg20311730 |  | -1.011 | 0.07 | -1.001 | 0.843 | 1.023 | | 0.000* |  |
| cg22578843 |  | -1.001 | 0.571 | -1.001 | 0.389 | 1.003 | | 0.015* |  |
| cg23884187 |  | -1.001 | 0.604 | 1.001 | 0.46 | 1.002 | | 0.212 |  |
| cg25350722 |  | -1.005 | 0.859 | 1.004 | 0.889 | -1.017 | | 0.501 |  |
| cg00223952 | NLRP11 | -1.004 | 0.105 | -1.007 | 0.002* | 1.003 | | 0.138 |  |
| cg00554118 |  | -1.005 | 0.525 | 1.006 | 0.459 | -1.121 | | 0.000* |  |
| cg01861389 |  | 1.002 | 0.612 | 1.003 | 0.352 | -1.005 | | 0.083 |  |
| cg14003035 |  | -1.006 | 0.498 | -1 | 0.979 | -1.012 | | 0.191 |  |
| cg14940165 |  | 1.003 | 0.445 | -1.003 | 0.377 | 1.005 | | 0.146 |  |
| cg16185840 |  | -1.002 | 0.585 | -1.001 | 0.873 | -1.012 | | 0.001* |  |
| cg16345566 |  | -1.053 | 0.317 | 1.066 | 0.215 | 1.004 | | 0.942 |  |
| cg17382459 |  | -1.001 | 0.905 | 1.011 | 0.114 | -1.024 | | 0.001* |  |
| cg17856544 |  | -1.012 | 0.008* | -1.006 | 0.164 | -1.057 | | 0.000* |  |
| cg18006085 |  | -1.004 | 0.114 | -1.003 | 0.237 | 1.002 | | 0.431 |  |
| cg21113318 |  | 1.001 | 0.945 | -1.024 | 0.028* | -1.01 | | 0.336 |  |
| cg25730098 |  | -1.024 | 0.000* | -1.012 | 0.012* | 1.007 | | 0.129 |  |
| cg04695373 | NLRP12 | -1.151 | 0.000* | -1.064 | 0.008* | 1.023 | | 0.318 |  |
| cg07042144 |  | -1.092 | 0.000* | -1.06 | 0.001* | 1.062 | | 0.000* |  |
| cg08703289 |  | -1.034 | 0.000* | -1.01 | 0.271 | -1.031 | | 0.001* |  |
| **cg22337438** |  | **-1.118** | **0.000*** | **-1.07** | **0.002*** | **1.069** | | **0.003*** |  |
| cg25866075 |  | -1.052 | 0.000* | -1.039 | 0.000* | 1.042 | | 0.000* |  |
| cg27188056 |  | -1.091 | 0.000* | -1.036 | 0.013* | 1.073 | | 0.000* |  |
| cg01424997 | NLRP13 | -1.017 | 0.004* | -1.001 | 0.876 | -1.011 | | 0.054 |  |
| cg03770907 |  | -1.02 | 0.178 | -1.01 | 0.522 | 1.084 | | 0.000* |  |
| cg08846272 |  | -1.014 | 0.129 | -1.001 | 0.907 | -1.09 | | 0.000* |  |
| cg24086536 |  | -1.011 | 0.006* | -1.002 | 0.653 | -1.01 | | 0.012* |  |
| cg00355992 | NLRP14 | -1.007 | 0.053 | -1.015 | 0.000* | 1.009 | | 0.011* |  |
| cg04346415 |  | 1 | 0.783 | -1.003 | 0.004* | 1.004 | | 0.000* |  |
| cg04997812 |  | 1.002 | 0.639 | 1.012 | 0.013* | -1.022 | | 0.000* |  |
| cg08558873 |  | -1.014 | 0.000* | -1.012 | 0.001* | 1.006 | | 0.071 |  |
| cg08757348 |  | -1.021 | 0.000* | -1.029 | 0.000* | 1.02 | | 0.000* |  |
| cg09371047 |  | -1.006 | 0.001* | -1.008 | 0.000* | 1.002 | | 0.319 |  |
| cg11679142 |  | -1.002 | 0.452 | 1.008 | 0.005* | -1.011 | | 0.000* |  |
| cg11838299 |  | -1.02 | 0.000* | -1.024 | 0.000* | 1.012 | | 0.005* |  |
| cg12471986 |  | -1.015 | 0.017* | -1.021 | 0.001* | 1.017 | | 0.006* |  |
| cg13219080 |  | -1.006 | 0.106 | -1.001 | 0.701 | 1.002 | | 0.671 |  |
| cg14948785 |  | -1.015 | 0.000* | -1.009 | 0.004* | -1.008 | | 0.011* |  |
| cg15125684 |  | -1.028 | 0.000* | -1.019 | 0.011* | 1.003 | | 0.687 |  |
| cg17228942 |  | -1.038 | 0.000* | -1.027 | 0.004* | 1.004 | | 0.665 |  |
| cg21873011 |  | -1.011 | 0.001* | -1.017 | 0.000* | 1.014 | | 0.000* |  |
| cg22235417 |  | -1.007 | 0.058 | 1.009 | 0.011* | -1.029 | | 0.000* |  |
| cg22443982 |  | -1.013 | 0.000* | -1.021 | 0.000* | 1.012 | | 0.000* |  |
| cg22867063 |  | -1.017 | 0.002* | -1.017 | 0.002* | 1.017 | | 0.002* |  |
| cg24113449 |  | -1.007 | 0.007* | -1.005 | 0.056 | 1.004 | | 0.076 |  |
| **cg01290568** | IL-1β | **-1.186** | **0.000*** | **-1.106** | **0.000*** | **1.114** | | **0.000*** |  |
| cg02596281 |  | -1.037 | 0.000* | -1.015 | 0.061 | 1.003 | | 0.676 |  |
| cg07935264 |  | -1.070 | 0.000* | -1.051 | 0.000* | 1.058 | | 0.000* |  |
| cg08332330 |  | 1.000 | 0.957 | -1.000 | 0.907 | -1.003 | | 0.050* |  |
| cg15836722 |  | -1.141 | 0.000* | -1.054 | 0.001* | 1.060 | | 0.000* |  |
| cg18773937 |  | -1.078 | 0.000* | -1.046 | 0.003* | 1.074 | | 0.000* |  |
| cg20157753 |  | -1.020 | 0.000* | -1.048 | 0.000* | 1.069 | | 0.000* |  |
| cg23149881 |  | -1.139 | 0.000* | -1.069 | 0.001* | 1.107 | | 0.000* |  |
| cg03304763 | IL-18 | -1.113 | 0.000* | -1.067 | 0.000* | 1.079 | | 0.000* |  |
| cg04100971 |  | -1.010 | 0.115 | 1.003 | 0.620 | -1.040 | | 0.000* |  |
| cg04929355 |  | -1.111 | 0.000* | -1.066 | 0.001* | 1.060 | | 0.002* |  |
| **cg05687149** |  | **-1.101** | **0.000*** | **-1.073** | **0.000*** | **1.073** | | **0.000*** |  |
| cg07267984 |  | -1.004 | 0.477 | 1.006 | 0.207 | -1.042 | | 0.000* |  |
| cg07809301 |  | -1.005 | 0.241 | 1.019 | 0.000* | -1.036 | | 0.000* |  |
| cg09122223 |  | -1.128 | 0.000* | -1.070 | 0.000* | 1.042 | | 0.019* |  |
| cg10439914 |  | 1.001 | 0.620 | -1.000 | 0.887 | 1.004 | | 0.002* |  |
| cg11304234 |  | -1.079 | 0.000* | -1.047 | 0.001* | 1.020 | | 0.129 |  |
| cg12756645 |  | -1.006 | 0.152 | 1.002 | 0.597 | -1.068 | | 0.000* |  |
| cg13591377 |  | -1.013 | 0.026* | 1.005 | 0.402 | -1.043 | | 0.000* |  |
| cg22399458 |  | -1.001 | 0.713 | 1.003 | 0.400 | -1.063 | | 0.000* |  |
| cg23831217 |  | -1.013 | 0.020* | -1.007 | 0.193 | -1.028 | | 0.000* |  |
| cg26534425 |  | -1.080 | 0.000* | -1.047 | 0.004* | 1.030 | | 0.054 |  |

KD1: Kawasaki disease before IVIG treatment; KD3: Kawasaki disease > 3 weeks after IVIG treatment; FC: febrile control; HC: healthy

control.
